# PR65A Phosphorylation Regulates PP2A Complex Signaling

**DOI:** 10.1371/journal.pone.0085000

**Published:** 2014-01-21

**Authors:** Kumar Kotlo, Yongna Xing, Sonia Lather, Jean Michel Grillon, Keven Johnson, Randal A. Skidgel, R. John Solaro, Robert S. Danziger

**Affiliations:** 1 Department of Medicine and Center for Cardiovascular Research, University of Illinois at Chicago, Chicago, Illinois, United States of America; 2 Department of Physiology, University of Illinois at Chicago, Chicago, Illinois, United States of America; 3 Jesse Brown Veterans Administration, Chicago, Illinois, United States of America; 4 Department of Oncology, University of Wisconsin, Madison, Wisconsin, United States of America; 5 Department of Pharmacology, University of Illinois at Chicago, Chicago, Illinois, United States of America; University of Iowa, United States of America

## Abstract

Serine-threonine Protein phosphatase 2 A (PP2A), a member of the PPP family of phosphatases, regulates a variety of essential cellular processes, including cell-cycling, DNA replication, transcription, translation, and secondary signaling pathways. In the heart, increased PP2A activity/signaling has been linked to cardiac remodeling, contractile dysfunction and, in failure, arrythmogenicity. The core PP2A complex is a hetero-trimeric holoenzyme consisting of a 36 kDa catalytic subunit (PP2Ac); a regulatory scaffold subunit of 65 kDa (PR65A or PP2Aa); and one of at least 18 associated variable regulatory proteins (B subunits) classified into 3 families. In the present study, three *in vivo* sites of phosphorylation in cardiac PR65A are identified (S303, T268, S314). Using HEK cells transfected with recombinant forms of PR65A with phosphomimetic (P-PR65A) and non-phosphorylated (N-PR65A) amino acid substitutions at these sites, these phosphorylations were shown to inhibit the interaction of PR65A with PP2Ac and PP2A holoenzyme signaling. Forty-seven phospho-proteins were increased in abundance in HEK cells transfected with P-PR65A versus N-PR65A by phospho-protein profiling using 2D-DIGE analysis on phospho-enriched whole cell protein extracts. Among these proteins were elongation factor 1α (EF1A), elongation factor 2, heat shock protein 60 (HSP60), NADPH-dehydrogenase 1 alpha sub complex, annexin A, and PR65A. Compared to controls, failing hearts from the Dahl rat had less phosphorylated PR65A protein abundance and increased PP2A activity. Thus, PR65A phosphorylation is an *in vivo* mechanism for regulation of the PP2A signaling complex and increased PP2A activity in heart failure.

## Introduction

Serine-threonine Protein phosphatase 2A (PP2A), a member of the PPP family of phosphatases, is abundant (up to 1% of total cellular protein), highly conserved from yeast to humans, and regulates a variety of essential cellular processes, including cell-cycling, DNA replication, transcription, and translation and multiple signaling pathways [1], [2]. Prominently, it regulates a number of genes, including FOXO1, which is involved in cell cycle control and apoptosis [3] and it has been identified as a tumor suppressor [4]. In the heart, PP2A activity/signaling regulates cardiac remodeling, contractility and, in failure, arrythmogenicity [5]–[7]. In heart failure, PP2A dephosphorylates phospholamban (PLN), which reduces sarcoplasmic reticulum (SR) Ca^2+^ ATPase (SERCA2a) activity, SR Ca^2+^ uptake, and SR Ca^2+^ loading [8]–[10]. PP2A also dephosphorylates cardiac troponin I (cTnI), which thereby increases myofilament sensitivity to Ca^2+^ [11]–[13].

The core PP2A complex is a hetero-trimeric holoenzyme consisting of a 36 kDa catalytic subunit (PP2Ac), which shares homology with other serine-threonine phosphatases, a regulatory scaffold subunit of 65 kDa (PR65A or PP2Aa) and one of at least 18 associated variable regulatory proteins (B subunits) classified into 3 families, i.e., B (B55), B′(B56) and B″, that bind to the core enzyme via the scaffold subunit and are generally believed to confer substrate specificity for dephosphorylation, cellular localization, and enzymatic activity [2], [14], [15]. In PP2A holoenzymes, PP2Ac and regulatory subunits specifically recognize the top ridge of huntingtin-elongation- A subunit-TOR (HEAT) repeats 11–15 and the N-terminal HEAT repeats of the A subunit [16]–[18] [19], [20] respectively. Fifteen HEAT repeats of the scaffold subunit hold the catalytic and regulatory B subunits together in a horseshoe. De Grande et al [21] have recently reported the detection of expression of 9 different (of 13 genes) regulatory subunits, a catalytic subunit, and two scaffolding proteins of PP2A in human heart. The expression pattern of the subunits varies across and within species as well as within cardiomyocytes and in heart failure.

We have recently reported that cardiac PR65A is phosphorylated and the phospho-protein abundance is decreased in heart failure [22]. The present studies were performed to determine the effect of PR65A phosphorylation on the PP2A complex and signaling.

## Results

### Identification of phosphorylated amino acids in cardiac PR65A (Fig 1)

**Figure 1:**
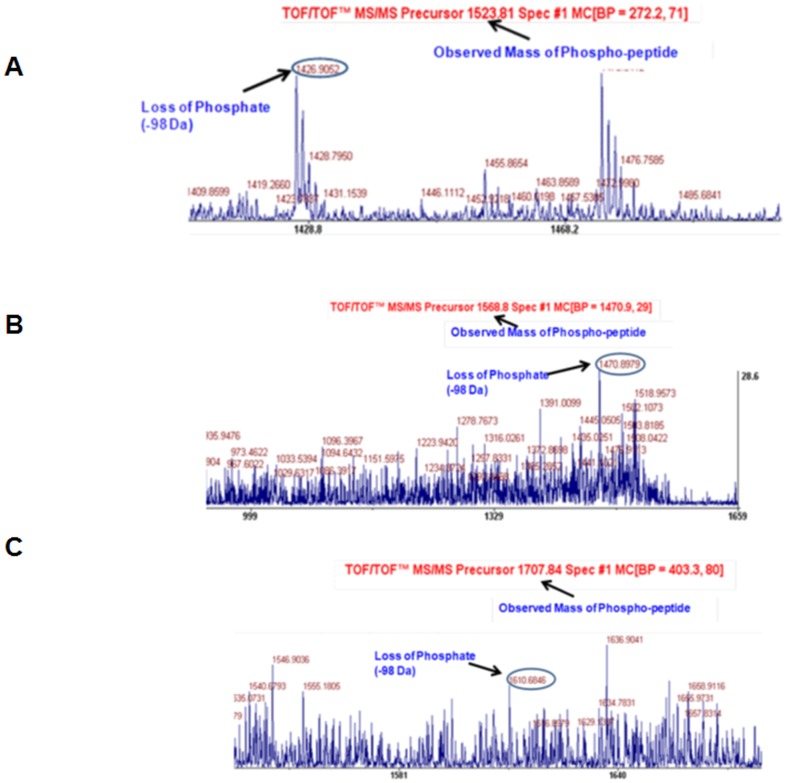
Identification of phosphorylated amino acids in cardiac PR65A. MALDI/TOF spectra for PR65A extracted from Dahl R rat hearts show that S303 (Panel A), T268 (Panel B), and S314 (Panel C) are phosphorylated. Methods: Phosphoproteins from control Dahl R rats were separated in 2D-DIGE. The protein spot corresponding to PR65A was excised from the gel, subjected to tryptic digestion and phosphopeptide enrichment using Supel-Tips (Sigma-Aldrich, Inc). The phosphopeptides were spotted on an eMALDI plate followed by mass spectrometry and database search. The spectra of all peptides were manually evaluated for the loss of phosphate which is shown in red circles and the mass and sequence of the peptides are shown. X-axis represents mass and Y axis represents intensity (see General Experimental Methods for details).

Phosphorylated amino acids in PR65A were identified in non-failing (normal) hearts from Dahl salt-resistant (R) rats. Phospho-enriched proteins extracted were separated by 2D-DIGE. The protein spot corresponding to PR65A, identified by its MW 65.28 and PI 5.0, was excised from the gel, subjected to tryptic digestion and phospho-peptides were enriched employing TiO_2_. The PR65A phospho-peptides were identified by MALDI-MS mass spectrometry and phosphorylated amino acids identified by MALDI-TOF. Three phosphorylated amino acids were identified, S303, T268, and S314. Each of these phosphorylation sites is conserved across the mouse, rat, and human proteins.

### Phosphorylation of PR65A reduces PP2A activity and its interaction with PP2Ac (Fig 2)

**Figure 2:**
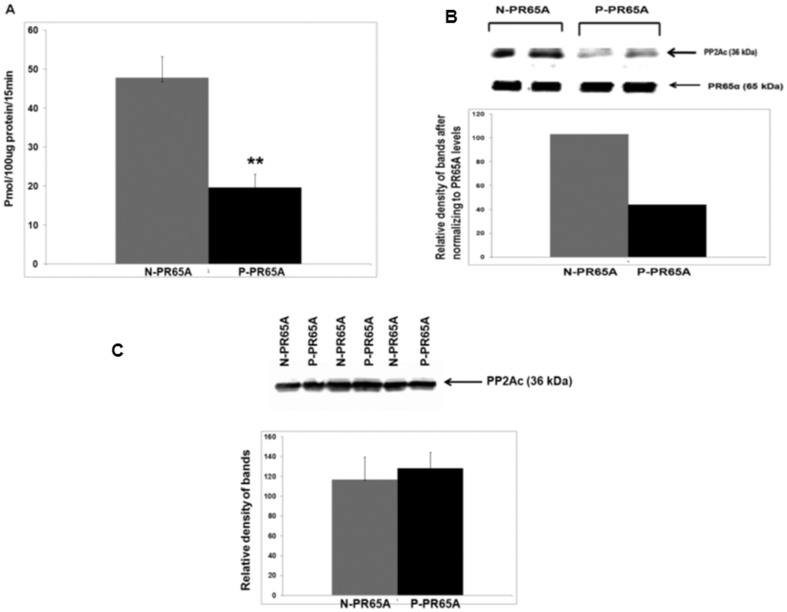
PP2A phosphatase activity and interaction of PR65A with PP2Ac is reduced by PR65A phosphorylation. Panel 2A: PP2A activity in immunoprecipitates of protein extracts from HEK cells transfected with recombinant N-PR65A vs. P-PR65A. N = 3; * P<0.01. Panel 2B: HEK cells were transfected with either N-PR65A or P-PR65A constructs immunoprecipitated with anti-PR65A polyclonal antibody (see General Experimental Methods) followed by Western blotting with anti-PP2Ac Ab and PR65A Ab. Panel 2C: HEK cells were transfected with either N-PR65A or P-PR65A constructs and protein extracts were immunoblotted with anti-PP2Ac Ab. (n = 3).

In order to study the effects of the identified PR65A serine/threonine phosphorylations on signaling, PP2A activity and PR65A interaction with PP2Ac were compared in HEK cells transfected with recombinant PR65A with phosphorylated amino acids mutated to alanines to eliminate phosphorylations (S303A, T268A, S314A)(N-PR65A) and recombinant PR65A with the amino acids mutated to glutamines to mimic phosphorylation (S303E, T268E,S314E)(P-PR65A). PP2A activity was less (P<0.01) in cells transfected with P-PR65A versus N-PR65A (Fig 2A). The effect of PR65A phosphorylation on its interaction with PP2Ac was examined in HEK cells transfected with either N-PR65A or P-PR65A by immunoprecipitation using anti-PR65A polyclonal antibody followed by Western blotting with monoclonal anti-PP2A catalytic subunit antibody (see Methods).Western blotting with anti-PR65A polyclonal antibody was performed on the same gel for loading control. Less PP2Ac was immunoprecipitated (Fig 2B) in cells transfected with P-PR65A versus N-PR65A, indicating that the phosphorylations reduced the interaction of PP2Ac with PR65A. No difference in PP2Ac expression was detected by Western analysis (Fig 2C).

### Phosphoprotein targets regulated by PR65A phosphorylation (Fig 3A, Table 1)

**Figure 3:**
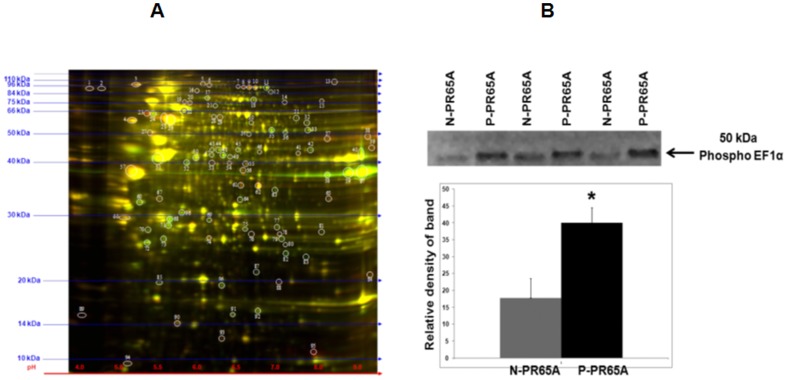
2D-DIGE Analysis of phosphoproteins from N-PR65A (green) vs. P-PR65A (red) transfected HEK cells. Panel A: Phospho-enriched protein samples from cells transfected with N-PR65A vs. P-PR65A were differentially labeled with Cydyes (N-PR65A = Cy5 red, P-PR65A = Cy2 green) and subjected to 2D-DIGE followed by phosphoprotein profiling (see General Methods). Molecular weight markers (kDa) are displayed to the left of each gel and pH markers are displayed underneath each gel. The spots are numbered according to size and PI. Spots 8, 23, 25, 28, 37, 38, 65, and 88 were identified. Panel B: Validation of increased phosphorylation of elongation factor-1 alpha by recombinant P-PR65A. Protein extracts from HEK cells transfected with N-PR65A and P-PR65A were immunoprecipitated with anti-phospho serine/threonine Ab followed by Western blotting with anti-rabbit monoclonal antibody against elongation factor 1 alpha. n = 3; * P<0.01.

In order to proximally link PR65A phosphorylation with cell protein phosphorylations and targets, phosphoproteins in HEK cells transfected with P-PR65A versus N-PR65A were profiled using 2D-DIGE analysis. Approximately 200 total phosphoprotein spots were resolved. Of these #60 and #37 were more and less, respectively, than 1.5-fold intense in cells transfected with P-PR65A versus N-PR65A. Eight proteins that were at least 2.2 fold more intense in cells transfected with recombinant P-PR65A versus N-PR65A were reasoned to be the most likely targets of PP2A and identified by MALDI MS (Table 1). Among these are elongation factors 1 alpha (EF1A) and 2, heat shock protein 60 (HSP60), NADPH-dehydrogenase 1 alpha sub-complex, annexin A, and PR65A. Increased phosphorylation of EF1A was confirmed using immunoprecipitations (Figure 3B).

**Table 1:** Phosphoprotein spots which demonstrated 2-fold or greater abundance in HEK cells transfected with P-PR65A vs. N-PR65A.

Spot#	Protein name	Accession#	MW	PI	Peptide count	Protein score	Total Ion score	Fold change
8	Elongation factor (EF2)	EF2_Human	95,277	6.4	40	856	100	2.3
23	Serine/Threonine Protein Phosphatase 2A 65 kDa regulatory subunit A alpha isoform	2AAA_Human	65,267	5.0	27	592	100	2.6
25	60 kDa heat shock protein, mitochondrial	CH60_Human	61.016	5.7	26	1010	100	2.9
88	NADH dehydrogenase [ubiquinone] 1 alpha subcomplex subunit 8	NDUA8_Human	20.092	7.6	9	402	100	2.4
28	Splicing factor U2AF 65 kDa subunit	U2AF2_Human	53,467	9.2	15	586	100	2.3
37	Elongation factor 1-alpha-1	EF1A1_Human	50,109	9.1	13	237	100	2.2
38	Putative elongation factor 1-alpha-like 3	EF1A3_Human	50,153	9.2	18	244	100	2.2
65	Annexin A1	ANXA1_Human	38,690	6.6	17	568	100	2.2

Identified by MALDI-TOF/TOF mass spectrometry (C.I.>99%) Fold-differences in abundance (P-PR65A vs. N-PR65A) (Change).

### Phosphorylated PR65A is less abundant and PP2A activity is increased in failing Dahl rat hearts (Fig 4)

**Figure 4:**
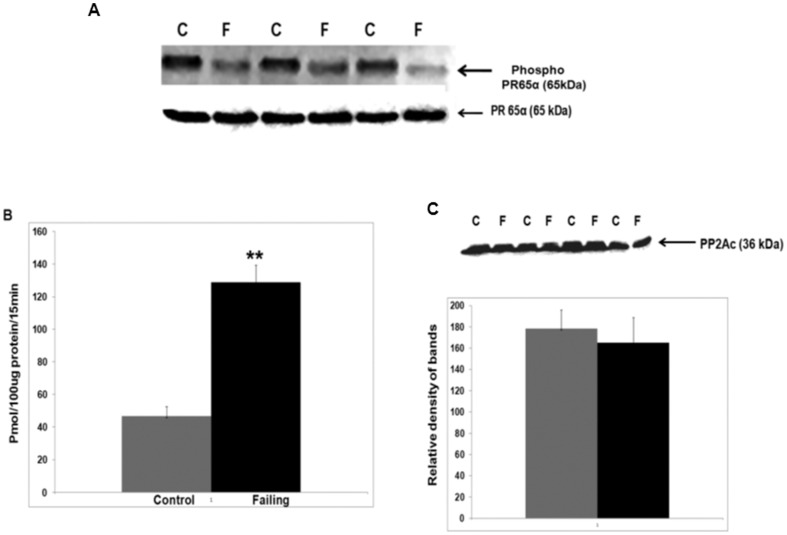
Reduced expression of phosphorylated PR65A and greater PP2A activity in systolic failing hearts. Panel A: Protein extracts from control (Dahl R) and (Dahl S) rat hearts were immunoprecipitated with monoclonal anti-phospho serine/threonine antibodies followed by Western blotting with monoclonal Ab against PR65A. n = 3 P<0.05. As a control protein extracts from control (Dahl R) and (Dahl S) rat hearts were subjected to SDS-PAGE followed by Western blotting with Ab against PR65A. Panel B: PP2A phosphatase activity in systolic failing hearts. PP2A activity in protein extracts from left ventricular walls of systolic failing (Dahl S) and control (Dahl R) rats, N = 4; *P<0.01. Panel C: Western blotting of protein extracts from control and failing rat hearts with Ab against PP2A catalytic subunit (n = 4).

Phosphorylated PR65A, measured in control (Dahl R) and failing Dahl (Dahl S) rat hearts by immunoprecipitation, was greater in Dahl R versus Dahl S hearts (Fig 4A P<0.05). PP2A activity, measured by dephosphorylation of the phosphopeptide (K-R-pT-I-R-R) with total protein extracts, was greater in failing than in control Dahl rat hearts (Fig. 4B). Western blotting did not show a difference in PP2Ac abundance (Fig 4C).

## Discussion

**Figure 5:**
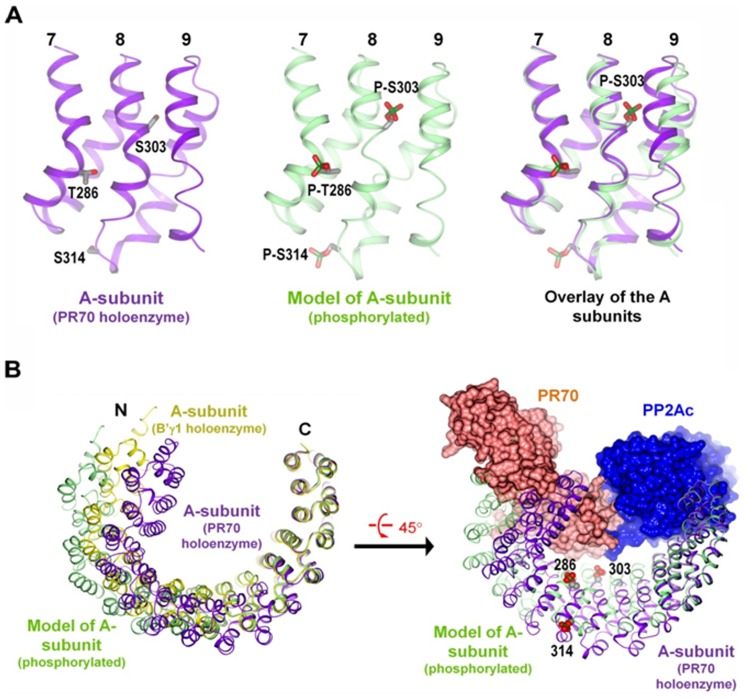
Proposed defect in PP2A holoenzyme assembly by phosphorylation of PR65A. (A) Phosphorylation and modeled changes in HEAT repeats 7, 8, 9 of the A-subunit. The A-subunit from the PR70 holoenzyme (left, purple), the modeled structure of the phosphorylated A subunit (middle, light green), and their overlay (right) are shown. Residues Thr268, Ser303, Ser314, and their phosphorylation counterparts are shown in cylinder and colored by atom type. (B) Alignment of the PR70 and B′γ1 holoenzymes and the model of the phosphorylated A subunit by HEAT repeats 11–15. The A subunits are in ribbon. PP2Ac and the PR70 regulatory subunit are in space fill. The A subunits from the PR70 and B′γ1 holoenzymes and the model of the phosphorylated A subunit are colored purple, yellow, and light green, respectively.

These studies show regulation of PP2A signaling *in vivo* by the phosphorylation of PR65A at S303, T268, and S314, via modulation of formation of the PP2A holoenzyme complex. Thr286, Ser303, and Ser314 are located at the concave surface and the bottom ridge of the A subunit and within HEAT repeats 7 and 8 (Figure 5A, left panel), suggesting that these residues do not directly contact PP2Ac or the regulatory subunits (Figure 5B, right panel). In modeling the structure of the phosphorylated A subunit based on the structure of the B′γ1 holoenzyme (PDB code: 2NPP), Ser303 is buried between HEAT repeats 8 and 9 in all active PP2A holoenzymes, suggesting. that significant structural shifts in HEAT repeat 9 and rearrangement of Ser303 side chain are required to expose the phosphorylated Ser 303 (P-S303) (Fig. 5A, middle and right panels), and to accommodate the phosphate group, thereby alleviating the repulsive interactions between the phosphate group and internal hydrophobic structures and, as a result, stabilize the A subunit in an open conformation that hinders formation of the compact A subunit conformations in the B′γ1 (PDB code: 2NPP) and PR70 (PDB codes: 4I5L, 4I5N) holoenzymes (Figure 5B, left panel) [23], [24]. This opened conformation of the A subunit would impede interaction of the PR70 regulatory subunit in the PR70 holoenzyme (Figure 5B, right panel). The reduced PP2A activity with P-PR65A versus N-PR65A is consistent with a recent observation suggesting that failure in formation or disruption of PP2A holoenzymes stabilizes of PP2Ac in a latent, inactive form [25].

Approximately sixty phosphoproteins were less abundant in cells transfected with N-PR65A versus P-PR65A. These are reasoned to be in vivo targets of PP2A in the HEK cells. Among those we identified are HSP60, a molecular chaperone which plays a protective role against stress-induced cardiomyocyte injury [26], [27], PkA-mediated phosphorylation of HSP60 has been shown to regulate histone 2B (H2B) chaperoning and association with the plasma membrane [28]. Another phosphoprotein spot in this group was Elongation Factor 1 α (EF1A). In the heart, EF2 phosphorylations have been linked to preserved energy charge during ischemia, delayed the development of ischemic contracture, and reduced myocardial apoptosis and necrosis [29]. We have also reported reduced phosphorylated EF2 in cardiac hypertrophy [22]. Annexin, an endogenous, glucocorticoid-regulated anti-inflammatory protein which has been specifically linked to the preservation of cardiomyocyte viability and post-ischemic cardiac function [30]–[32], was also identified in this group. Phosphorylation of annexin A1 at Ser5 by TRPM7 kinase prevents the formation of a critical alpha-helix for interaction with both membranes and S100A11 protein [33]. Annexin has also been shown to regulate the phosphorylation of phospholipase A2 in mast cells. These identified phospho-proteins did not interact with PP2Ac in studies recently published by Herzog et al [34] (personal communication) and reinforce the need to complement the identification of protein-protein interactions with signaling studies. There are several possible explanations for the discrepancy. They did not do a PR65A pull-down, suggesting that PR65A may interact with other proteins. Our results suggest that proteins we identified would require the dephosphorylated state of PR65A for maximal interaction with the PP2A holoenzyme complex. It is thus possible that PR65A was in the phosphorylated state in the studies of Herzog et al [34]. Also, a stringent filtering procedure to separate true interactors from contaminating proteins was used in their studies. Thus the substrates that we identified may bind to PP2A with low affinity and thus the interaction was not preserved during the washing-procedure of the pull-downs. An additional possibility is that the increased phosphorylation of our candidates might be an indirect effect, e.g., mutated PR65A might affect a downstream phosphatase or kinase and leading to increased phosphorylation levels of the phosphoproteins that we identified independent of PP2A direct effects. Importantly, we reason that some phosphoprotein changes are not detected by the methodology used because their abundance is below detection and/or their affinity for the anti-serine/threonine phosphate Ab used for phospho-enrichment is very low.

Approximately 37 phospho-proteins were noted on 2D-DIGE images to be more abundant in cells transfected with N-PR65A versus P-PR65A. These are likely indirectly regulated by PP2A, perhaps through ‘activating’ dephosphorylations of kinases [35]. These were not identified in this study.

**Figure 6:**
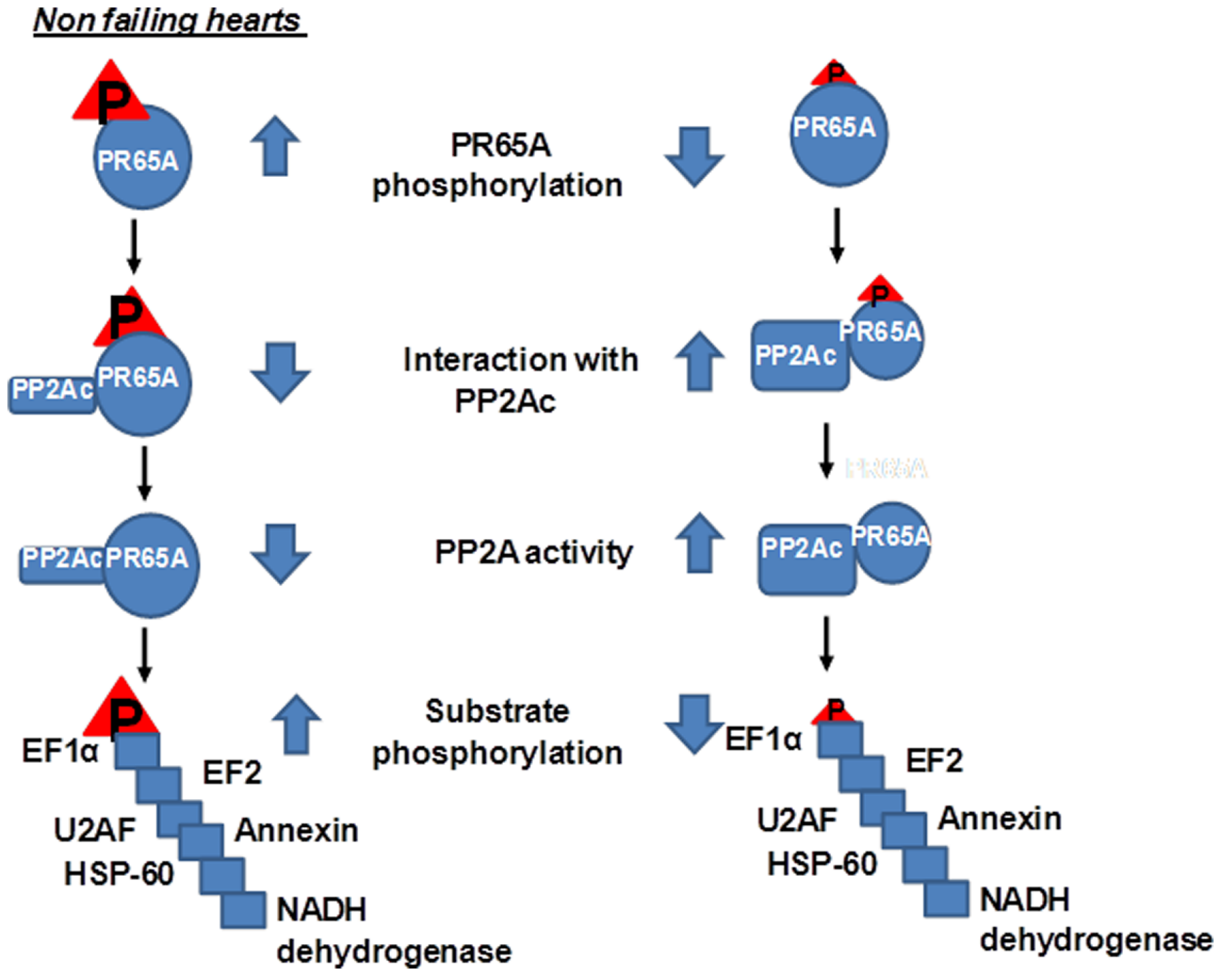
Schematic of signaling elucidated in present work.

Based on our results we believe that reduced phosphorylation of PR65A in failing hearts increases its interaction with PP2Ac and PP2A phosphatase activity. This leads to decreased phosphorylation of proteins such as EF1A, EF2, HSP-60, U2AF, annexin, and NADH dehydrogenase (Figure 6).

The regulation of PR65A by phosphorylation adds to the increasingly complex picture of cellular PP2A signaling, which not only includes post-translational, but also transcriptional and translational changes to multiple PP2A subunits [21]. This work adds to the evolving insight into the complex regulation of phosphatases in general. Additionally, various combinations of phosphorylations of the subunits of the PP2A holoenzyme have contrasting and complex effects on its activity in that phosphorylation of B56δ by protein kinase A has been shown to activate PP2A [36]

## Methods

University of Illinois Institutional Animal Care and Use Committee (IACUC) and ethics committee approved this study.

### Materials and supplies

HEK cells were purchased from ATCC. Antibodies against PR65a and PP2A catalytic subunit were from Santa Cruz Inc and Millipore Inc. Antibody against EF1A was from Cell signaling Inc. PP2A activity kit was purchased from Millipore Inc. QuickChange™ mutagenesis kit was from Stratagene.

Male 250–300 g Dahl salt-sensitive (S) or Dahl salt-resistant (R) rats were purchased from Harlan Sprague Dawley. All animal studies were done in accordance with University of Illinois at Chicago Institutional Animal Care and Use Committee (IACUC). Rats were maintained on a 8% NaCl diet ad lib.

### Identification of phosphorylated amino acids in PR65A

PR65A phosphoprotein was resolved from phospho-enriched protein homogenates of left ventricle tissue by 2Dl gel electrophoresis, the corresponding protein spot was excised from the gel and subjected to tryptic digestion followed by tandem mass spectrometry analysis.

The PR65A spot was picked up by an Ettan Spot Picker (Amersham BioSciences, Piscataway, NJ) based on the in-gel analysis. The gel spot was washed 2–3 times then digested in-gel with modified porcine trypsin protease (Trypsin Gold, Promega, and Fitchburg, WI). The digested tryptic peptides were subjected to Supel-Tips (Sigma-Aldrich, St. Louis, MO) to enrich phosphopeptides, following the manufacturer's protocol. The phosphopeptides were desalted by Zip-tip C18 (Millipore, Inc., Billerica, MA). Peptides were eluted from the Zip-tip with 0.5 µl of matrix solution (*α*-cyano-4-hydroxycinnamic acid (5 mg/ml in 50% acetonitrile, 0.1% trifluoroacetic acid, 25 mM ammonium bicarbonate)) and spotted on the matrix-associated laser desorption/ionization (MALDI) plate.

### Mass Spectrometry (MS)

The protein accession number was submitted to UCSF Protein Prospector (http://prospector.ucsf.edu/prospector/mshome.htm) to run a virtual digest. All precursors containing predicted phosphorylation sites were manually entered into the inclusion list for MSMS analysis. MALDI-TOF MS and TOF/TOF tandem MS/MS were performed on an AB SCIEX TOF/TOF™ 5800 System (AB SCIEX, Framingham, MA). MALDI-TOF mass spectra were acquired in reflectron positive ion mode, averaging 4000 laser shots per spectrum. TOF/TOF tandem MS/MS fragmentation spectra were acquired on all precursors containing predicted phosphorylation sites, plus the 5 most abundant ions present in each sample (excluding trypsin autolytic peptides and other known background ions).

### Database search

Both the resulting peptide mass and the associated fragmentation spectra were submitted to MASCOT search engine to search NCBI. Searches were performed without constraining protein molecular weight or isoelectric point, with variable carbamidomethylation of cysteine, oxidation of methionine residues and phosphorylation of serine, threonine, tyrosine, and with one missed cleavage also allowed in the search parameters. Candidates with either protein score C.I. % or ion C.I. % greater than 95 were considered significant. The spectra of all peptides containing potential phosphorylation sites were manually evaluated for the loss of phosphate.

### PR65α constructs

Full-length PR65α DNA construct (Origene Inc) was used to introduce S/T to A (*T268A, S303A, and S314A*) and S/T to E mutations (*T268E, S303E, and S314E*) by site-directed mutagenesis using the QuikChange™ mutagenesis kit (Stratagene, La Jolla, CA) according to the manufacturer's protocols. All constructs were sequenced by the DNA sequencing core facility at University of Illinois at Chicago prior to use in the experiments.

### Transfections

HEK cells were transfected with recombinant PR65α constructs for 48 hrs using lipofectamine 2000 (Invitrogen) according to the manufacturer's instructions. After 48 hrs, protein extracts were prepared for immunoprecipitation and PP2A activity assay studies.

### Immunoprecipitation

Cell lysates from either heart tissue or HEK cells transfected with PR65A and sds22 constructs with S/T to A and S/T to E substitutions were incubated overnight at 4°C with PR65A regulatory subunit antibodies (Santa Cruz Biotechnology, Inc.) with constant shaking. Fifty µl of protein A/G agarose beads were added to each reaction and incubated with constant shaking at 4°C for 2 hrs. The samples were spun down for 1 min and the pelleted beads were washed sequentially with PBS with 1% Triton-X-100, PBS with 0.5% Triton-X-100, and PBS with 0.1% Triton-X-100. The pellets were re-suspended in SDS sample buffer and subjected to Western blotting with monoclonal antibodies against catalytic subunits of PR65A (Millipore, Inc.).

### Phosphatase activity assay

PP2A was immunoprecipitated with monoclonal antibodies against the catalytic subunit (anti–PP2Ac subunit clone 1D6 from Millipore, Inc) followed by incubation with a phosphorylated peptide substrate for 15 min. Then malachite green solution was added and the phosphate released measured by the color development at 650 nM. Assay kits available from Millipore, Inc. PP2A activity was measured as the difference between phosphatase activity in the presence and absence of okadaic acid (5 nM added 15 min prior to substrate).

### Phosphoprotein profiling

Phosphoprotein analysis was performed using 2-D DIGE using CyDye staining and MALDI-MS for identification as previously described [22]. Image scans were carried out immediately following the SDS-PAGE using Typhoon TRIO (Amersham BioSciences) following the protocols provided. The scanned images were analyzed by Image QuantTL software (GE-Healthcare), and then subjected to in-gel analysis and cross-gel analysis using DeCyder software version 6.5 (GE-Healthcare). The ratio change of the protein differential expression was obtained from in-gel DeCyder software analysis.

### MALDI-TOF/TOF Mass Spectrometry

Based on the fold changes in the phosphoprotein abundance, protein spots chosen for analysis were excised by Ettan Spot Picker (GE Healthcare) and washed multiple times to remove staining dye and other inhibitory chemicals. Gel spots were dried and then rehydrated in digestion buffer containing sequencing grade modified trypsin. Proteins were digested in-gel at 37°C and digested peptides were extracted from the gel with TFA extraction buffer and shaking. The digested tryptic peptides were desalted using C-18 Zip-tips (Millipore) and then mixed with CHCA matrix (alpha-cyano-4-hydroxycinnamic acid) and spotted into the wells of a MALDI plate. Mass spectra (MS) of the peptides in each sample were obtained using an ABSciex 4700 Proteomics Analyzer and ten to twenty of the most abundant peptides in each sample were further subjected to fragmentation and tandem mass spectrometry (MS/MS) analysis. The combined MS and MS/MS spectra were submitted for database search using GPS Explorer software equipped with the MASCOT search engine to identify proteins from the NCBI non-redundant protein database. Proteins that were identified with a confidence interval (C.I.) greater than 99% are reported.

### Modeling of phosphorylated PR65A

The structure of the A subunit (PR65A) in the B′γ1 holoenzyme (PDB code: 2NPP) was used as the starting model for the phosphorylated PR65A. The phosphate groups were added to residues Thr286, Ser303, and S314 of the A subunit (B′γ1 holoenzyme), and changes required for accommodating the phosphate groups were modeled. Structural modeling was performed and energy minimized using Sybyl (Tripos). The model with minimum changes was used for structural comparison with the A subunit from different PP2A holoenzymes (B′γ1 and PR70 holoenzymes).

### Rat hearts

Methods for control and failing rat heart models used have previously been described [22]. Systolic heart failure criteria were either 1) FS<0.40 or 2) signs of distress, e.g., tachypnea, loss of appetite, weight loss.

### Statistical analysis

Values are expressed as the mean ± SD. Differences among different treatments were determined by one way ANOVA for repeated measurements. Results are considered significant at P<0.05.
